# Editorial: The neural economy hypothesis: Changes with aging and disease to cones and other central nervous system visual neurons

**DOI:** 10.3389/fnagi.2022.1054455

**Published:** 2022-11-22

**Authors:** Ann E. Elsner, Adam M. Dubis, Jessica I. W. Morgan, Ferenc B. Sallo

**Affiliations:** ^1^School of Optometry, Indiana University, Bloomington, IN, United States; ^2^Global Business School for Health, University College London, London, United Kingdom; ^3^Scheie Eye Institute, Department of Ophthalmology, University of Pennsylvania, Philadelphia, PA, United States; ^4^Hôpital Ophtalmique Jules-Gonin, University of Lausanne, Lausanne, Switzerland

**Keywords:** cones, macula, risk to photoreceptors, vascular changes, fovea, cone distribution, retinal degeneration, diabetic retinopathy

Photoreceptors and other central nervous system cells are considered post-mitotic and at their maximum numbers before birth, although the human retina undergoes considerable developmental changes in the first few years of life (Hendrickson and Yuodelis, 1984). In the human fovea, the packing of cones becomes denser at the central fovea by migration, not by new cones being formed. Cone outer segments elongate by adding more segments over time, and the inner retinal cells move to more eccentric locations, reducing light scatter to enhance foveal acuity. To provide decades of vision these neurons and their networks must have inherent repair processes and be supported and renewed by glial and other cells. There are limitations to the extent of support available to visual system neurons, especially photoreceptors with their high metabolic rates, the assault by incident light on the retina, and the need for optical clarity that constrains the numbers and locations of blood vessels. Further, these support systems can fail with aging and disease, creating a harsh microenvironment for photoreceptors.

Cones have a remarkable ability to survive, compared with rods. Alterations in cone structure and function enable them to stay connected in their circuitry to provide at least some vision. This is the neural economy hypothesis, i.e., that neurons adapt to their environment and can modify their physiology to be economical (Elsner et al., 2020). Improving the microenvironment in aging and disease has the potential to preserve cones and visual system circuitry, thereby preserving vision.

Many possible changes to visual system neurons over the life span include cones reverting to having fewer outer segments, and even existing as mainly cell bodies as can be found in eyes with missing or damaged retinal pigment epithelium such as in age-related macular degeneration (Sarks et al., 1988), myopia (Jonas et al., 2013), or glaucoma (Dichtl et al., 1998). However, cone photopigment can increase to more normal levels over time following a retinal detachment (Elsner et al., 1992).

New technologies enable *in vivo* assessments of photoreceptor health with the added advantages of including younger subjects with earlier stage disease and longitudinal studies of disease progression. Histology for many human diseases such as age-related macular degeneration is often weighted toward old eyes and end-stage disease. This Research Topic inspects photoreceptors, their microenvironment, and visual pathways in health, disease, and aging, using a variety of techniques including histology, *in vivo* imaging, and function measures in both human eyes and animal models.

There are extreme differences in the changes in the retina when the initial insult is a genetic mutation as opposed to a metabolic challenge provided by aging or diseases such as diabetes. The effect of aging on the retina is spatially heterogeneous, as described in Zouache: the greater prominence of aging changes occur in the regions with the highest energy requirements. The environment of neurons changes more in the macula than in the periphery, and in the outer retina and choroid as compared to the inner retina. In one family of progressive retinal degenerations due to genetic defects, known as retinitis pigmentosa, damage to cones over time and space can be complex because of the viability factor provided by another neuron, the rods (Roberts). In a mathematical model for progressing cone degeneration caused by a reduction in rod derived cone viability factor (RdCVF), a simple formula is insufficient as both the amount of RdCVF produced and the amount of RdCVF the cones require to survive will vary with retinal location in different manners.

Longer term degeneration of cones is also described by Li et al., who evaluated long term cone structure in the inherited retinal dystrophy Oligocone Trichromacy. This condition results in decreased but usually stable cone function. Their work demonstrates that even though cone function is reduced, cones survive, shown by multimodal cellular imaging.

A more complex structure to function relationship was observed by Jolly et al., who evaluated the changes in color vision in patients with choroideremia. Their work shows that there is early loss of tritan (blue-yellow) loss, before loss of red-green discrimination. While the disease progresses, both chromatic axes had functional loss, even while visual acuity and OCT based structural parameters suggest that the retina remained intact. Their work suggests that the redundancy in the acuity system is likely maintained longer.

The structure to function relationship is also studied for aging by Baraas et al. looking at the first detected loss of red-green function and using the visual acuity of perifoveal L-cones. Their work confirms cone density did not decrease significantly in early age-related macular degeneration, through direct visualization of photoreceptors, even in areas with decreased L-cone visual function. Cones in patients with age-related macular degeneration had significantly shortened outer segments. Using visual function methods, the detection rate of photons by cones is proposed by Braham chaouche et al. to detect age-related macular degeneration. Using multi-modal imaging methods, Yuhas et al. propose that damage to cone axons may be detected by the combined use of directional OCT and scanning laser polarimetry or other polarization-sensitive imaging methods, demonstrating early changes to photoreceptor integrity in the central macula as a harbinger to ocular or to neurological disease.

Elsner et al. report that diabetic patients with vessel remodeling do not have a large decrease of cones and surprisingly have retinal thickness on OCT within normal limits. There is a greater variability in the reflectance and distributions of cones. Significantly low cone density is found in some patients, including younger patients, but the large variability of older controls makes difficult isolating metabolic damage.

Large changes with aging in more central neurons are described by Fiuza et al. for the rat non-image forming thalamus. The daily variations in neurons of calretinin expression that are important for calcium buffering and signaling decline progressively with age, with effects not restricted to particular regions suggesting this is a generalized process.

Taken together, these papers show how cones and other visual neurons survive, but with altered function. Imaging and function studies provide the potential for early detection and streamlining of treatment trials.

## Author contributions

AE organized the topic review, wrote the initial call for papers, invited editors, edited manuscripts, contributed one manuscript to the topic, and led writing of the editorial. AD invited two editors, served as an editor and reviewer, contributed one manuscript to the topic, and contributed to the initial draft of the editorial. JM edited the call for papers, edited manuscripts, and contributed to the initial draft of the editorial. AE, AD, JM, and FS approved the submitted version.

## Funding

The preparation of this article was supported by research grants from the AE National Eye Institute (EY024315 to FS), National Eye Institute (EY028601 and EY030227 to JM), and the NIHR Biomedical Resource Centre to Moorfields Eye Hospital NHS Trust and UCL Institute of Ophthalmology to AD.

## Conflict of interest

JM's lab receives financial support from AGTC. AE is the CEO and founder of Aeon Imaging. The remaining authors declare that the research was conducted in the absence of any commercial or financial relationships that could be construed as a potential conflict of interest.

## Publisher's note

All claims expressed in this article are solely those of the authors and do not necessarily represent those of their affiliated organizations, or those of the publisher, the editors and the reviewers. Any product that may be evaluated in this article, or claim that may be made by its manufacturer, is not guaranteed or endorsed by the publisher.
